# Identification, Phylogeny, and Transcript of Chitinase Family Genes in Sugarcane

**DOI:** 10.1038/srep10708

**Published:** 2015-06-02

**Authors:** Yachun Su, Liping Xu, Shanshan Wang, Zhuqing Wang, Yuting Yang, Yun Chen, Youxiong Que

**Affiliations:** 1 Key Laboratory of Sugarcane Biology and Genetic Breeding, Fujian Agriculture and Forestry University, Ministry of Agriculture, Fuzhou 350002, China

## Abstract

Chitinases are pathogensis-related proteins, which play an important role in plant defense mechanisms. The role of the sugarcane chitinase family genes remains unclear due to the highly heterozygous and aneuploidy chromosome genetic background of sugarcane. Ten differentially expressed chitinase genes (belonging to class I~VII) were obtained from RNA-seq analysis of both incompatible and compatible sugarcane genotypes during *Sporisorium scitamineum* challenge. Their structural properties and expression patterns were analyzed. Seven chitinases (*ScChi*I1, *ScChi*I2, *ScChi*I3, *ScChi*III1, *ScChi*III2, *ScChi*IV1 and *ScChi*VI1) showed more positive with early response and maintained increased transcripts in the incompatible interaction than those in the compatible one. Three (*ScChi*II1, *ScChi*V1 and *ScChi*VII1) seemed to have no significant difference in expression patterns between incompatible and compatible interactions. The ten chitinases were expressed differentially in response to hormone treatment as well as having distinct tissue specificity. *ScChi*I1, *ScChi*IV1 and *ScChi*VII1 were induced by various abiotic stresses (NaCl, CuCl_2_, PEG and 4 °C) and their involvement in plant immunity was demonstrated by over-expression in *Nicotiana benthamiana*. The results suggest that sugarcane chitinase family exhibit differential responses to biotic and abiotic stress, providing new insights into their function.

Sugarcane, *Saccharum* spp., is an established source of sugar and has become the current benchmark renewable feedstock for efficient biofuel production. Plant disease is an important factor that affects the sugarcane yield and quality. Sugarcane smut (*Sporisorium scitamineum*) is one of the main diseases in sugarcane production areas^1^. Commercial sugarcane cultivars are poly-aneuploid interspecific hybrids with ploidy level ranging from 5X to 16X^2^ and contain in excess of a hundred chromosomes^3^. Pyramiding resistance using cross breeding, in combination to the important agronomic traits of stalk yield, sucrose content and disease resistance in a specific variety is very difficult. It is generally believed that the cultivation of disease-resistant cultivars is the most economic and effective measure to prevent smut^4^. Characterising sugarcane disease resistance genes will benefit to resistance breeding by providing excellent genetic resources and provide the basis for development of related molecular markers for sugarcane breeding.

Research on sugarcane pathology has focused on the cytology^5^, morphology^6^, physiology and biochemistry^7^, as well as genetics of sugarcane smut, to explore resistant mechanisms. Although the genome sequencing of *S. scitamineum* should provide new insights into the pathogenic mechanisms of sugarcane smut^8^, the studies on the interaction between sugarcane and *S. scitamineum* at the molecular level focuses mainly on the cloning and quantification of resistance-related genes, such as flavonoid pathway transcription factors X1, serine/threonine protein kinase, auxin-binding protein and 1-aminocyclopropane-1-carboxylate oxidase, in sugarcane genotypes after inoculation with *S. scitamineum*^9^,^10^. Transcriptomics can help to reveal the impact of *S. scitamineum* on sugarcane, such as the metabolic pathways and networks of molecular regulation to explore key resistant genes responding to *S. scitamineum* attack^11^.

Pathogenesis-related (PR) proteins are specific proteins induced by pathological conditions and play an important role in the plant disease resistance reaction^12^. Chitinases (EC 3.2.2.14), are one category of PR protein, and can catalyze chitin in the main components of the pathogen cell wall, then inhibit the growth of fungi and help to improve plant defense against fungi^13^,^14^,^15^. Plant chitinase genes have been grouped into seven classes (class I~VII), suggesting that chitinase isozymes were encoded by a multi-gene family^16^,^17^,^18^. A variety of chitinase family genes involved in pathogen attack^19^,^20^, hormone application^21^, temperature change^22^, high salt content^22^, metal and wounding stresses^23^, have been cloned and characterized from various plants such as *Arabidopsis thaliana*^24^, *Nicotiana tabacum*^25^, *Oryza sativa*^26^, *Triticum aestivum*^17^ and *Sorghum bicolor*^27^. A class I chitinase isolated from *Hordeum vulgare* has been reported to demonstrate antifungal activity^28^. A pathogen-inducible acidic class III chitinase protein, was purified from *N. tabacum* infected with tobacco *mosaic virus* (TMV)^20^. Genomic sequences of chitinases in *Vitis vinifera* were isolated by PCR walking^29^. Two of these belong to the class I chitinases with a putative vacuolar (*Vvchit1a*) and extracellular (*Vvchit1b*) localization, while the third one belongs to class III (*VvchitIII*)^29^. Singh *et al.*^17^ characterized an acidic form of class VII chitinase (glycosyl hydrolase family 19) from *T. aestivum* and demonstrated that the purified chitinase exerted a broad-spectrum antifungal activity against *Alternaria* sp., *Colletotrichum falcatum*, *Fusarium* sp., *Rhizoctonia solani*, *Sarocladium oryzae* and *Pestalotia theae*. Liu *et al.*^21^ described a chitinase gene *Mmchi1* in *Mikania micrantha* and demonstrated that its transcripts were up-regulated after challenged with *Cuscuta campestris*. The expression levels of *Mmchi1* gene were also increased in response to abscisic acid (ABA), salicylic acid (SA), zinc sulfate (ZnSO_4_) and wounding. Davis *et al.*^30^ tested the expression of various chitinases (class I, II and IV) of *Ananas comosus* in response to pathogen-associated signals, including necrotrophic pathogen *Fusarium subglutinans* f. sp. *pini*, SA and jasmonic acid (JA) stresses.

Plant chitinases distribute in plant roots, stems, leaves, and flowers^31^. Many chitinase genes are developmentally regulated and may play a part in the specific physiological processes^32^. The expression of *AtchitIV* gene was analyzed in *Arabidopsis* exposed to abiotic stress by Gerhardt *et al.*^23^. Transcripts accumulation was detected in leaves in response to UV light exposure, exogenous SA administration and wounding. The *AtchitIV* expression was also analyzed during *Arabidopsis* embryo development^23^.

In sugarcane, chitinase genes have been cloned and identified by their individual classes^22^,^33^,^34^. Ten nucleotide sequences of sugarcane chitinases are currently found in the GenBank database (GU219846.1, KF664180.1, KF279662.1, KF279661.1, EF123043.1, EU914816.1, EU914815.1, EF120468.1, EF113913.1, AF402937.1). Among these, only three full-length chitinase genes, *ScChi* (KF664180), *ScChi*VII1 (KF279662) and *ScChiB1* (EU914815.1) have been functionally analyzed^22^,^33^,^34^. A class III chitinase gene *ScChi*, highly expressed in sugarcane leaf and stem epidermal tissues, has been cloned and characterized^22^. Its transcript was more abundant and maintained higher level for longer in a resistance cultivar challenged with *S. scitamineum*. Overexpression of *ScChi* in *N. benthamiana* suggested a close relationship between the expression of *ScChi* and plant immunity. Wang *et al.*^34^ reported a class VII chitinase gene *ScChi*VII1 from sugarcane. Differential gene expression pattern of *ScChi*VII1 in the smut resistant genotype Yacheng05-179 compared with the susceptible genotype Liucheng03-182 was found when challenged with *S. scitamineum*. Expression of *ScChi*VII1 in buds was significantly higher than that in roots, stems, leaves and epidermis. Rahul *et al.*^33^ obtained a full-length class IV chitinase gene *ScChiB1* from the red rot resistant cultivar Co93009 with a high expression level in the incompatible interaction during challenge with *Colletotrichum falcatum* compared with the compatible interaction. In addition, a partial chitinase sequence from chewing cane cultivar Fuan was up-regulated after challenged with *C. falcatum*^35^.

In this study, chitinase genes in sugarcane responding to *S. scitamineum* were surveyed and comparative analysis was conducted. The transcriptomes of Yacheng05-179 (smut resistant) and ROC22 (smut susceptible) challenged with *S. scitamineum* at 24 h, 48 h and 120 h post infection (hpi) were used to identify differentially expressed genes encoding chitinases. The structure and the classification of 10 sugarcane chitinase family genes (class I~VII), as well as their response to pathogens, defense signal compounds and their expression in different sugarcane tissues were investigated using qRT-PCR (quantitative real-time polymerase chain reaction). In addition, the full-length cDNA sequences of three chitinase genes, *ScChi*I1, *ScChi*IV1 and *ScChi*VII1, were isolated and further analyzed for subcellular localization, transient expression in *N. benthamiana*, and gene expression profile under various abiotic stresses.

## Results

### Expression profile of sugarcane chitinase genes after *S. scitamineum* inoculation

The transcriptome analysis of sugarcane induced by *S. scitamineum* at 24 hpi, 48 hpi and 120 hpi was conducted, using the *S. scitamineum*-resistant and -susceptible genotypes (Yacheng05-179 and ROC22). An unigene library containing a total of 99,824 unigenes was constructed^11^.

We previously reported identification and functional analysis of two sugarcane chitinase genes, *ScChi* (KF664180)^22^ and *ScChi*VII1 (KF279662)^34^. To identify more chitinase genes, from the transcriptome analysis, 17 unigenes differentially expressed during sugarcane-*S. scitamineum* interaction were identified as possibly encoding for chitinase genes (Table 1). Transcripts of chitinase genes (gi34957207, Sugarcane_Unigene_BMK.68059, gi35992663, Sugarcane_Unigene_BMK.60821, Sugarcane_Unigene_BMK.56580, Sugarcane_Unigene_BMK.64954, Sugarcane_Unigene_BMK.48857, gi36021860, Sugarcane_Unigene_BMK.60821) were all up-regulated in the resistant genotype Yacheng05-179. However, 12 genes displayed a mixed expression pattern (10 up-regulated and 2 down-regulated) in the susceptible genotype ROC22. Furthermore, the expression of chitinase genes in Yacheng05-179 (24 hpi ~ 48 hpi) occurred earlier than that in ROC22 (48 hpi ~ 120 hpi), suggesting resistance specificity and early timing of these genes in the incompatible interaction between sugarcane and *S. scitamineum*.

### Phylogenetic analysis of chitinase gene family

**Figure 1:**
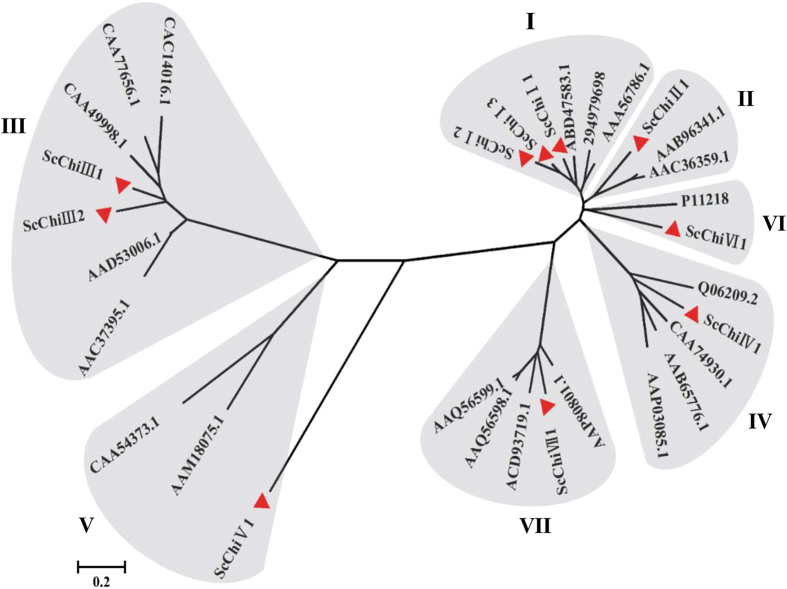
The phylogenic tree of chitinase family genes in sugarcane and other plant species. The tree was constructed using the neighbor-joining method and diagrams drawn with MEGA 5.05. The red triangle represents the 10 sugarcane chitinase family genes.

Among the 17 differentially expressed chitinase unigenes, a total of 9 members was predicted to have full-length sequences with open reading frames (ORFs). The assembled sequence of *ScChi*VII1 based on homologous cloning method according to the predicted *S. bicolor* chitinase gene (XM_002460419.1) was added. To study the phylogenetic relationships of the chitinase family genes in sugarcane, a multiple alignment analysis was performed. The 10 genes with ORF structures were classified into seven types (class I ~ VII) based on the similarity of their amino acid sequences with 21 biotic stress resistance-related chitinases of other plant species from NCBI^17^,^18^. As shown in Fig. 1, they were segregated into two branches, one comprising classes III and V, and the other one including the classes I, II, IV, VI and VII. The 10 sugarcane chitinase genes were named by classification system of the chitinase in the phylogenetic tree and described as *ScChi*I1 (gi32815041), *ScChi*I2 (gi34957207), *ScChi*I3 (Sugarcane_Unigene_BMK.68059), *ScChi*II1 (gi35992663), *ScChi*III1 (Sugarcane_Unigene_BMK.51590), *ScChi*III2 (Sugarcane_Unigene_BMK.60821), *ScChi*IV1 (Sugarcane_Unigene_BMK.56580), *ScChi*V1 (Sugarcane_Unigene_BMK.64954), *ScChi*VI1 (Sugarcane_Unigene_BMK.48857) and *ScChi*VII1. The nucleotide and protein sequences of these chitinases are provided in Supplementary Table S1.

### Sequence analysis of chitinase gene family

**Figure 2:**
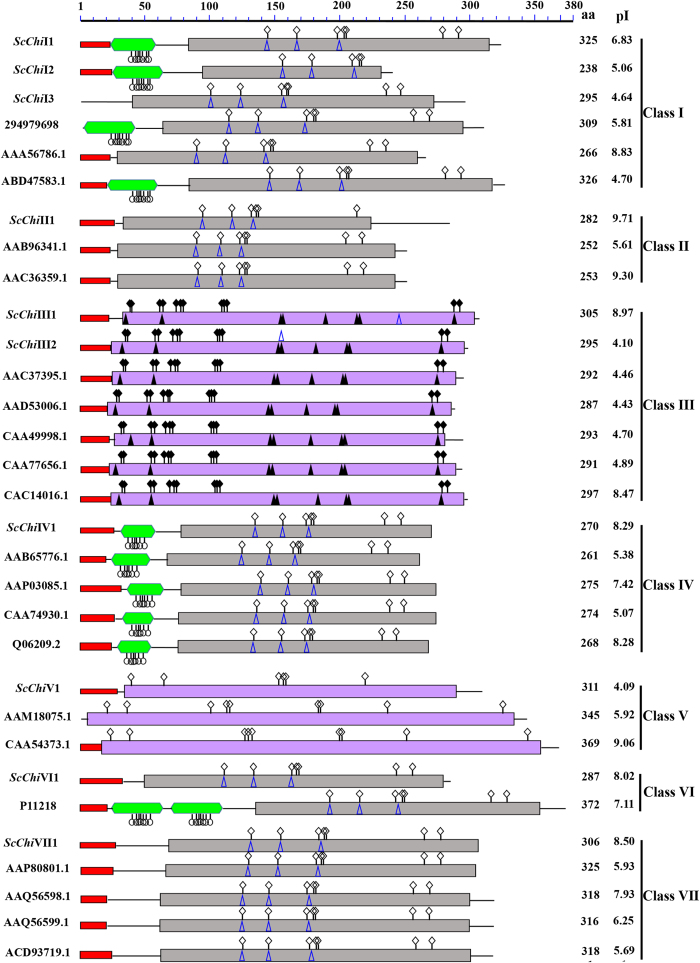
Domain architecture of chitinases classes I~VII in sugarcane and other plant species. aa: the number of amino acids; pI: isoelectric point; 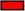: signal peptide; 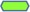: chitin binding domain (CBD); 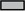: glycoside hydrolase family 19 chitinase domain; 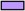: glycoside hydrolase family 18 chitinase domain; 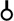: carbohydrate binding site; 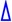: catalytic residues; 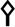: sugar binding site; 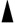: active site; 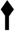: substrate-binding cleft.

In order to gain insight into the diversification among the above 10 chitinases from sugarcane and 21 from other plant species, several features of the predicted proteins were analyzed. The typical domains of chitinase, including chitin binding domain (CBD), chitinase domains of glycoside hydrolase family 18 and family 19, were shown in Fig. 2. In addition, the signal peptide, isoelectric point (pI) and the number of amino acids (aa) were also presented in Fig. 2. We found that not all chitinases contained signal peptide at their N-termini, such as *ScChi*I3 and *O. Sativa* chitinase (294979698) in class I and *Momordica charantia* chitinase (AAM18075.1) in class V. The length of the ORFs in sugarcane chitinases ranged from 238 aa to 325 aa. The average ORF length was 291 aa. The isoelectric point (pI) in different members was not identical in the same class, as some were acidic and others were basic. The sugarcane chitinases, including classes I, II, IV, VI and VII members, have a lysozyme-like domain in their structures which may exhibit lysozyme activity.

Class I members *ScChi*I1 and *ScChi*I2 both contained the N-terminal signal peptide, following the chitin binding domain (CBD) which was rich in cysteines (9) and a glycoside hydrolase family 19 chitinase domain. Though the protein domain of *ScChi*I3 lacked a signal peptide and the CBD structure and was different from those of *ScChi*I1 and *ScChi*I2, they sharing 63.96% amino acid sequence identity. A spacer hinge region, rich in proline (3, 12 and 13) and glycine (5, 6 and 5) residues, was found between the CBD and the glycoside hydrolase family 19 chitinase domains of *ScChi*I1, *ScChi*I2 and *ScChi*I3. Class II chitinase *ScChi*II1 lacked the CBD and the hinge region, but contained a N-terminal signal peptide and a glycoside hydrolase family 19 chitinase domain (amino acids 34 ~ 223), sharing a high degree of homology (70.82%) with class I members. Like class I protein, class IV chitinase *ScChi*IV1 consisted of the CBD, hinge region and glycoside hydrolase family 19 chitinase domain. However, there was only 59% identity in the catalytic domain among class I and class IV. The class VI chitinase *ScChi*VI1, which lacked the duplicated CBDs in its N-terminal region which was different from chitinase (P11218) in *Urtica dioica* endochitinase^36^, had a signal peptide, a hinge region (1 prolines and 6 glycines) and a glycoside hydrolase family 19 chitinase domain. Class VII chitinase *ScChi*VII1 lacked the CBD and the hinge region, and its amino acid sequences were 47.57% homology to class I and Class II chitinases. Unlike other sugarcane chitinase family members, the catalytic domain in class III (*ScChi*III1 and *ScChi*III2) and class V (*ScChi*V1) chitinases was glycosyl hydrolase family 18 but not glycoside hydrolase family 19. These results suggest that all ORFs in sugarcane chitinases contained at least one typical domain.

### Tissue-specific expression of chitinase family genes in sugarcane

**Figure 3:**
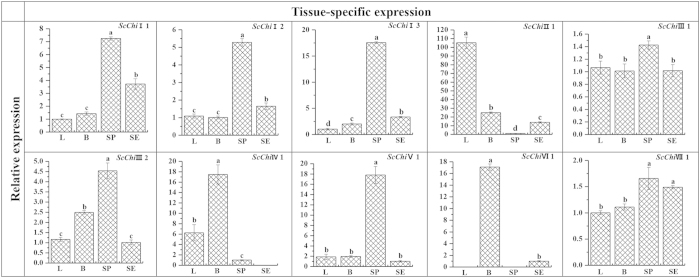
Tissue-specific expression analysis of the 10 chitinase family genes in different tissues of sugarcane genotype Yacheng05-179 by qRT-PCR. Data was normalized to the *GAPDH* expression level. All data points are the means ± SE (n = 3). Different lowercase letters indicate a significant difference, as determined by the least-significant difference test (p-value < 0.05). L: Leaf; B: Bud; SP: Stem pith; SE: Stem epidermis.

qRT-PCR was performed to determine the expression patterns of these putative chitinase genes in different sugarcane above-ground tissues. As shown in Fig. 3, the expression of chitinase genes belonging to classes I, II, III, V and VII was detected in all of the four sugarcane tissues including leaf, bud, stem pith and stem epidermis. Compared with the other three tissues, the chitinase genes with the highest expression levels in stem pith were *ScChi*I1, *ScChi*I2, *ScChi*I3, *ScChi*III1, *ScChi*III2, *ScChi*V1 and *ScChi*VII1. *ScChi*II1 showed the highest level of transcripts in sugarcane tissues with transcripts most abundant in leaf. Transcripts of *ScChi*IV1 and *ScChi*VI1 accumulated to the highest level in bud tissues. These results showed a certain degree of tissue specificity in sugarcane chitinase family genes (Fig. 3).

### Accumulation of chitinase gene mRNAs in sugarcane post inoculation with *S. scitamineum*

**Figure 4:**
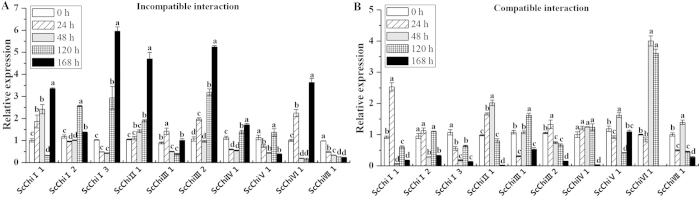
Expression analysis of the 10 chitinase family genes during sugarcane-smut (*Sporisorium scitamineum*) interaction by qRT-PCR. Data was normalized to the *GAPDH* expression level. All data points (with the deduction of their mocks) were the means ± SE (*n* = 3). Different lowercase letters indicate a significant difference, as determined by the least-significant difference test (*p*-value < 0.05). Incompatible interaction: the interaction between sugarcane resistant genotype Yacheng05-179 and *S. scitamineum*; Compatible interaction: the interaction between sugarcane susceptible genotype ROC22 and *S. scitamineum*.

qRT-PCR was used to examine the expression patterns of the 10 sugarcane chitinase family genes during sugarcane-smut interaction (Fig. 4). It was seen that all 10 transcripts were induced by infection of *S. scitamineum* but different patterns were evident.

During the incompatible interaction using Yacheng05-179, one smut resistant sugarcane genotype, early transcriptional elevation of *ScChi*I1, *ScChi*III1, *ScChi*III2 and *ScChi*VI1 was observed at 24 hpi (Fig. 4A). The transcript of *ScChi*III1 reached the maximum at 24 hpi, while the maximal accumulation of the other 3 genes was observed at 168 hpi. *ScChi*I2 and *ScChi*V1 transcripts decreased at 24 hpi and 48 hpi, but increased to the peak at 120 hpi and again reduced at 168 hpi. Although the *ScChi*I3 and *ScChi*IV1 accumulation decreased at initial stage (from 0 hpi to 48 hpi), they gradually elevated at the later stage (from 120 hpi to 168 hpi). *ScChi*II1 was up-regulated from 48 hpi to 168 hpi. In contrast, *ScChi*VII1 demonstrated a down-regulation during the incompatible interaction.

During the compatible interaction using ROC22, a popular genotype which is susceptible to *S. scitamineum*, transcripts of *ScChi*I1, *ScChi*II1 and *ScChi*III2 were observed to be elevated as early as 24 hpi, suggesting rapid response to the infection of smut pathogen (Fig. 4B). Their expression values were accumulated to the maximal levels at either 24 hpi or 48 hpi. Transcripts of *ScChi*I2 and *ScChi*IV1 maintained almost at the same level after inoculation. *ScChi*I3 was down-regulated compared with that at 0 hpi. The transcripts of *ScChi*III1, *ScChi*V1, *ScChi*VI1 and *ScChi*VII1 peaked at 48 hpi. The data indicated that all genes except *ScChi*VI1 had the lowest expression level at 168 hpi during the compatible interaction.

### Gene expression in response to different defense-related signal compounds

**Figure 5:**
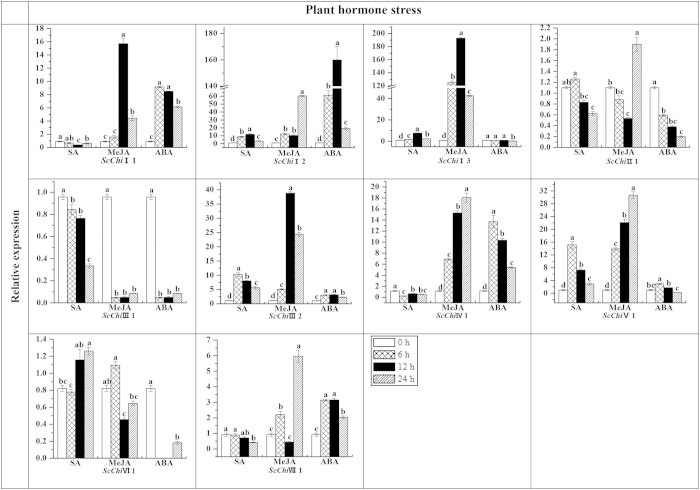
Expression analysis by qRT-PCR of the 10 chitinase family genes in Yacheng 05-179 plantlets after treatment with 5 mM SA, 25 μM MeJA and 100 μM ABA. Data was normalized to the *GAPDH* expression level. All data points were the means ± SE (*n* = 3). Different lowercase letters indicate a significant difference, as determined by the least-significant difference test (*p*-value < 0.05). SA, salicylic acid; MeJA, methyl jasmonate; ABA, abscisic acid.

Transcript accumulation of chitinase genes in sugarcane plantlets under different phytohormone treatments, including SA, MeJA (methyl jasmonate) and ABA stresses, were examined by qRT-PCR (Fig. 5). The results revealed that all three signal compounds up-regulated *ScChi*I2, *ScChi*III2 and *ScChi*V1, while *ScChi*III1 was down-regulated. *ScChi*I1, *ScChi*IV1 and *ScChi*VII1 were up-regulated by MeJA and ABA but down-regulated by SA. In addition *ScChi*VI1 was down-regulated by MeJA and ABA but up-regulated by SA. *ScChi*I3 was up-regulated by SA and MeJA but suppressed by ABA. ABA treatment down-regulated *ScChi*II1 while *ScChi*VII1 was up-regulated. These results suggest that the transcription of individual chitinase genes respond differently to SA, MeJA and ABA.

### Functional characterization of three chitinase genes, *ScChi*I1, *ScChi*IV1 and *ScChi*VII1, during pathogen infection

**Figure 6:**
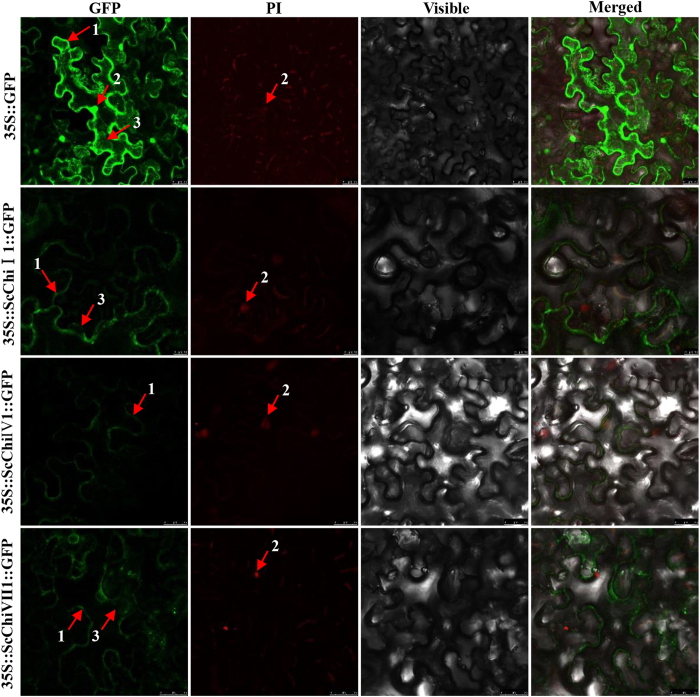
Subcellular localization analysis of ScChiI1, ScChiIV1 and ScChiVII1 in *Nicotiana benthamiana* leaves 48 h after infiltration. PI images indicate nuclear staining. The epidermal cells were used for taking images of green fluorescence, red fluorescence, visible light and merged light. Read Arrows 1, 2 and 3 indicated plasma membrane, nucleus and cytoplasm, respectively. Bar = 25 μm.

Based on the information of differentially expressed chitinase genes post *S. scitamineum* infection, the full-length cDNA sequences of three chitinase genes, *ScChi*I1, *ScChi*IV1 and *ScChi*VII1, were isolated from sugarcane. The sequence data of *ScChi*I1, *ScChi*IV1 and *ScChi*VII1 were submitted to GenBank under accession number of KF664182, KF664178 and KF664179, respectively. The ORF fragment was recombined into the plant expression vector of pCAMBIA 2300 containing the *35S* promoter and the *GFP* reporter gene. Their subcellular localization was characterized by transient expression of the target gene and GFP in *N. benthamiana* leaves with *Agrobacterium*-mediated transformation method^37^. Infiltrated leaves observed under a confocal laser scanning microscope showed that 35S::ScChiI1::GFP, 35S::ScChiIV1::GFP and 35S::ScChiVII1::GFP fusion proteins were located in cytoplasm and plasma membrane, plasma membrane, cytoplasm and plasma membrane, respectively (Fig. 6). In addition, the mock of 35S::GFP was shown in the nucleus, cytoplasm and plasma membrane cells.

**Figure 7:**
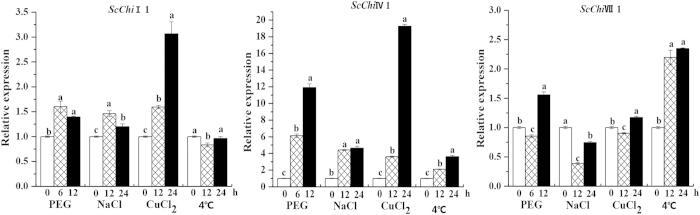
Expression analysis by qRT-PCR of *ScChi*I1, *ScChi*IV1 and *ScChi*VII1 genes in Yacheng05-179 plantlets after treatment with 25% PEG, 250 mM NaCl, 100 μM CuCl_2_ and low temperature (4 °C). Data were normalized to the *GAPDH* expression level. All data points were the means ± SE (n = 3). Different lowercase letters indicated a significant difference, as determined by the least-significant difference test (p-value < 0.05). PEG, polyethylene glycol; NaCl, sodium chloride; CuCl_2_, copper chloride.

Chitinase genes have been reported to be induced not only by biotic but also by abiotic stress^12^,^38^. The expression patterns of *ScChi*I1, *ScChi*IV1 and *ScChi*VII1 in Yacheng05-179 plantlets were investigated after treatment with 25% PEG (polyethylene glycol), 250 mM NaCl (sodium chloride), 100 μM CuCl_2_ (copper chloride), and low temperature (4 °C) (Fig. 7). This showed induction of high levels of *ScChi*IV1 transcripts with all four abiotic treatments. PEG, NaCl and CuCl_2_ appeared to cause an increase of accumulated *ScChi*I1 transcripts post stress, while low temperature caused slightly decrease at 12 h. The expression of *ScChi*VII1 was up-regulated by low temperature and down-regulated by NaCl. In response to PEG and NaCl stresses, the level of *ScChi*VII1 transcript reduced slightly at 6 h and 12 h, but increased at 12 h and 24 h, respectively.

### Transient expression of *ScChi*I1, *ScChi*IV1 and *ScChi*VII1 induces a defense response in *N. benthamiana*

**Figure 8:**
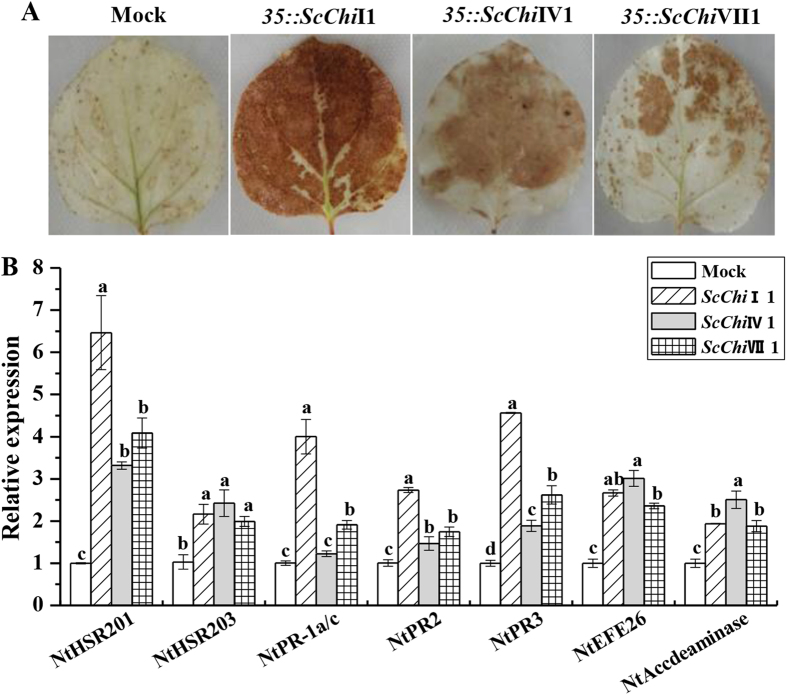
The transient expression of *ScChi*I1, *ScChi*IV1 and *ScChi*VII1 and qRT-PCR analysis of the immunity associated marker genes in *Nicotiana benthamiana* leaves. (**A**) DAB (3,3’-diaminobenzidinesolution) staining with *N. benthamiana* leaves 48 h after *Agrobacterium* strain infiltration. (**B**) The transcripts analysis of the immunity associated marker genes, including the hypersensitive response marker genes *NtHSR201* and *NtHSR203*, the jasmonate associated genes *NtPR-1a/c* and *NtPR2* and *NtPR3*, and the ethylene synthesis depended genes *NtEFE26* and *NtAccdeaminase. NtEF1-α* was used to normalize the transcript levels. Mock: the *Agrobacterium* strain carrying *35S::00*. All data points are the means ± SE (*n* = 3). Different lowercase letters indicate a significant difference, as determined by the least-significant difference test (*p*-value < 0.05).

To test whether the target genes can induce hypersensitive response (HR) and immunity in plant, *ScChi*I1, *ScChi*IV1 and *ScChi*VII1 genes were transiently over-expressed in *N. benthamiana* leaves. After 48 h post infiltration, a typical HR symptom with deeper DAB staining was found in the leaves expressing *35S::ScChi*I1, *35S::ScChi*IV1 and *35S::ScChi*VII1, respectively (Fig. 8). The bronzing color after over-expressing *ScChi*I1 was the darkest. Furthermore, the expression levels of seven immunity associated marker genes including the HR marker genes *NtHSR201* and *NtHSR203*, the JA- associated genes *NtPR-1a/c* and *NtPR2* and *NtPR3*, and the ethylene synthesis depended genes *NtEFE26* and *NtAccdeaminase*, were increased post 24 h infiltration. These results suggest that *ScChi*I1, *ScChi*IV1 and *ScChi*VII1 were involved in cell death responses.

## Discussion

Many plants contain multiple chitinase isozymes. They have been categorized into seven classes (class I ~ VII) based on their primary structure, substrate specificity, mechanisms of catalysis and sensitivity to inhibitors^17^,^18^. On the basis of the annotations of *O. sativa* and *Arabidopsis* genomic sequences, 37 and 24 chitinases were found in *O. sativa* and *Arabidopsis*, respectively^24^. Analysis revealed that each cluster had distinct amino acid characteristics. Krishnaveni *et al.*^39^ had observed three antifungal chitinases, CH1, CH2 and CH3, from *S. bicolor*. Four cDNAs encoding acidic and basic isoforms of chitinases were isolated from *Cladosporium fulvum*-infected tomato leaves^40^. We have previously reported cloning and identification of one class III and one class VII chitinases from sugarcane post *S. scitamineum* inoculation^22^,^34^. The current study of the sugarcane chitinase family indicated the presence of at least 17 expressed genes induced by smut pathogen.

Chitinase isozymes are a diverse group of enzymes with different characteristics, such as enzymatic activities, primary sequence, pI and cellular localization^41^. Based on the domain architecture of chitinases classes I~VII in sugarcane and other plants, not all chitinases contained a signal peptide, and the CBD structure was absent in *ScChi* VI1 (class VI). According to the most popular classification system described earlier^17^,^18^,^42^, class I chitinases contain three domains: a cysteine-rich N-terminal CBD, a proline- and glycine-rich hinge region and a highly conserved C-terminal catalytic domain. Class II chitinases are generally extracellular which lack the CBD and the hinge region, but their amino acid sequences in the catalytic domain are nearly identical to class I chitinases (more than 65%). Class III lacks CBD and has little sequence identity to the class I and class II catalytic domain, while Class IV contains the CBD, hinge region and catalytic domain, but displays deletion in the catalytic domain. Class V chitinases has little sequence similarity with the other chitinases, but more similar to bacterial chitinases, such as those from *Bacillus circulans* and *Serratia marcescens*. Class VI chitinases possess the duplicated CBDs in their N-terminal regions, while Class VII chitinases lack the CBD and the hinge region. In this study, seven types of sugarcane chitinases coincided with the former classification^17^,^18^,^42^. In Fig. 1, although *ScChi*V1 and the chitinase proteins from *Momordica charantia* (AAM18075.1) and *N. tabacum* (CAA54373.1) were not at the consistent branch of the phylogenic tree, it was assigned to the class V subfamily containing the same domain of glycoside hydrolase family 18. Nearly all sugarcane chitinases, except *ScChi*I 3, contained the N-terminal targeting domain which may involve in directing them to either the vacuole or the apoplast (Fig. 2). Like other plant species^43^, sugarcane chitinases of classes I, II, IV, VI and VII have the glycoside hydrolase family 19 domain belong to class PR-3 family, and class III and class V possess the glycoside hydrolase family 18 domain belong to PR-8 and PR-11 families, respectively. Chitinases including class I, II, IV, VI and VII were predicted to contain a lysozyme like domain^44^, suggesting that most sugarcane chitinases possess lysozyme activity.

According to previous reports, the only route of invasion of the smut pathogen is via sugarcane buds^45^. Previous studies also revealed that plant chitinases are developmentally regulated, indicating a role in the specific physiological processes^18^,^46^. In this study, transcripts of sugarcane chitinase genes differently accumulated in the noninfected sugarcane above-ground tissues (Fig. 3). Seven chitinase genes expressed at high expression levels in stem pith, suggesting specific roles in stem pith. *ScChi*II1 showed the highest expression level in sugarcane and its transcript was most abundant in leaf. Considering the significantly higher expression of *ScChi*IV1 and *ScChi*VI1 in sugarcane buds than in other tissues, it suggests that *ScChi*IV1 and *ScChi*VI1 may play a positive role in sugarcane smut resistance.

In the present study, during *S. scitamineum* infection (0 hpi ~ 168 hpi), the expression of at least 10 sugarcane chitinases was induced. However they showed different expression patterns in the incompatible/compatible interactions. In Yacheng05-179, four chitinase genes, *ScChi*I1, *ScChi*III1, *ScChi*III2 and *ScChi*VI1, rapidly responded to smut pathogen inoculation at initial stage (from 0 hpi~24 hpi) (Fig. 4), and reached maximal accumulation at 168 hpi. Conversely, in ROC22, almost all the target genes (except *ScChi*VI1) had lower expression levels at 168 hpi (Fig. 4). These results suggest that sugarcane chitinase genes are pathogen-inducible and are involved in disease resistance. Previously, a class III sugarcane chitinase gene *ScChi* was shown to be induced after challenge in the incompatible interaction (Yacheng05-179 *vs. S. scitamineum*) and its expression remained higher than that in a compatible interaction (Liucheng03-182 *vs. S. scitamineum*)^22^.

In plants, levels of chitinases are regulated by biotic and abiotic stress, such as pathogen infection, cold, drought, heavy metals, salt, and plant hormones^12^,^22^,^38^. As reported, SA, JA and ethylene are considered as the defense signal compounds for systemic acquired resistance (SAR) and induced systemic resistance (ISR), two types of plant induced resistance^21^. In plant responses to environmental stress, the reaction of the signaling molecule JA is the fastest, and plays an important part in resistance reaction. JA-related gene expression has been reported to be up-regulated and cause JA accumulation under biotic and abiotic stress^47^. Previous studies suggested that ABA affects plant response to biotic stress mainly via interaction with other stress responsive pathways^48^. In our study, the expression levels of sugarcane chitinase genes could be differentially modulated by SA, MeJA and ABA (Fig. 5). Exogenously applied SA resulted in an increase accumulation of *ScChi*I2, *ScChi*I3, *ScChi*III2, *ScChi*V1 and *ScChi*VI1 transcripts. Application of MeJA increased the expressions of *ScChi*I1, *ScChi*I2, *ScChi*I3, *ScChi*II1, *ScChi*III2, *ScChi*IV1, *ScChi*V1 and *ScChi*VII1. The exogenous application of ABA increased the levels of *ScChi*I1, *ScChi*I2, *ScChi*III2, *ScChi*IV1, *ScChi*V1 and *ScChi*VII1 transcripts.

Full-length cDNA sequences of three sugarcane chitinase genes, each one of class I chitinase *ScChi*I1, class IV chitinase *ScChi*IV1 and class VII chitinase *ScChi*VII1, were isolated from smut resistant genotype Yacheng05-179. These three genes were pathogen-inducible post *S. scitamineum* infection (Fig. 4), and were up-regulated by MeJA and ABA but down-regulated by SA (Fig. 5). Protein localization revealed that 35S::ScChiI1::GFP and 35S::ScChiVII1::GFP fusion proteins were located in cytoplasm and plasma membrane, while 35S::ScChiIV1::GFP was located in plasma membrane (Fig. 6). *ScChi*I1 and *ScChi*IV1 were up-regulated by PEG, NaCl and CuCl_2_ stresses, while *ScChi*VII1 was not (Fig. 7). *ScChi*IV1 and *ScChi*VII1 transcripts were increased under 4 °C low temperature stress, but *ScChi*I1 was not (Fig. 7). However, all these genes induced defense responses in *N. benthamiana* by transient expression (Fig. 8). These results suggest that the different sugarcane chitinases have individual functions in response to various environmental stresses.

Although functions of sugarcane chitinases genes are not fully understood, some chitinases in plant species have been shown to inhibit the growth of chitin-containing fungi, both *in vitro*^49^ and *in vivo*^14^,^15^. When compared with wild-type plants, in many cases, transgenic plants constitutively expressing chitinases showed enhanced resistance to fungal infection or delayed development of disease symptoms^50^,^51^. The transgenic *Musa acuminata* expressing the *O. sativa* chitinase gene exhibited resistance to black leaf streak disease caused by the pathogenic fungus, *Mycosphaerella fijiensis*^26^. In our previous work, a close relationship between the expression of sugarcane class III chitinase gene *ScChi* (KF664180) and plant immunity was demonstrated from inoculation experiments and the validation of *in vitro* antibacterial activity. There was also a report of smut resistance improvement in sugarcane varieties ROC22 and ROC10 by introduction of a β-1,3 glucanase together with the modified class I chitinase gene from *N. tabacum*^52^. From the characteristics of the 10 sugarcane chitinase genes obtained here, the possible contribution of all these genes for plant defense against pathogen attack is suggested. However, the conclusive validation and precise functional determination of these genes by genetic transformation into sugarcane is still in progress.

## Methods

### Plant materials and inoculation with *S. scitamineum*

Sugarcane varieties, Yacheng05-179 and ROC22, as well as smut whips, were obtained from the Key Laboratory of Sugarcane Biology and Genetic Breeding, Ministry of Agriculture (Fuzhou, China). Two-bud sets of both sugarcane genotypes (Yacheng05-179 and ROC22), were grown at 28 °C in condition of 12 h light/12 h dark photoperiod, then inoculated with 0.5 μL suspension containing 5 × 10^6^ spores·mL^−1^ of *S. scitamineum* in 0.01% (v/v) Tween-20. The controls were mock inoculated with 0.01% (v/v) Tween-20 in sterile distilled water instead of spores^37^ to eliminate the effect of wounding. At 0 hpi, 24 hpi, 48 hpi, 120 hpi and 168 hpi, one biological replicate consisting of five buds for each group were excised, immediately frozen in liquid nitrogen and then stored at −80 °C.

### Tissue distribution study

For tissue distribution study, one biological replicate with six healthy 10 month old plant of Yacheng05-179 was selected. The samples were collected from the youngest fully expanded leaf (+1 leaf) with a visible dewlap (the collar between the leaf blade and sheath), buds, stem pith and stem epidermis. These samples were fixed in liquid nitrogen and kept at −80 °C until RNA extraction^37^.

### Abiotic stress treatments

To investigate the expression of sugarcane chitinase family genes in response to stress factors, 4 month old tissue cultured plantlets of Yacheng05-179 were grown in water for one week and then exposed to various chemical stimuli^37^. The plantlets were treated with 5 mM SA solution, 25 μM MeJA in 0.1% (v/v) ethanol and 0.05% (v/v) Tween-20, 100 μM ABA, or 25% PEG8000, respectively. Plantlets were kept in conical tubes at 28 °C in condition of 16 h light/8 h dark photoperiod and harvested at 0 h, 6 h, 12 h and 24 h. Another group of sugarcane plantlets were separately treated with 250 mM NaCl, 100 μM CuCl_2_ and 4 °C low temperature for 0 h, 12 h, 24 h and 48 h. All of the above treatments were carried out in three biologic replicates.

### RNA extraction

Total RNA was extracted with Trizol reagent (Invitrogen, China) according to the manufacturer’s protocol. RNA was treated with DNase I (Promega, USA) to remove DNA contamination. The first-strand cDNA synthesis was completed by the Prime-Script^TM^ RT Reagent Kit (TaKaRa, China).

### Isolation of chitinase genes in sugarcane challenged with *S. scitamineum*

To isolate chitinase family genes in sugarcane post *S. scitamineum* infection, 26 unigenes which were differently expressed in Yacheng05-179 and ROC22 after inoculation with *S. scitamineum,* were annotated to chitinase genes^11^. Further BLAST by amino acid sequences, 17 unigenes predicted to encode for chitinase protein were analyzed. These unigenes ID were gi32815041, gi34957207, Sugarcane_Unigene_BMK.68059, gi35992663, Sugarcane_Unigene_BMK.51590, Sugarcane_Unigene_BMK.60821, Sugarcane_Unigene_BMK.56580, Sugarcane_Unigene_BMK.64954, Sugarcane_Unigene_BMK.48857, gi36021860, Sugarcane_Unigene_BMK.60821, gi35081719, Sugarcane_Unigene_BMK.49423, gi36003099, gi35980761, Sugarcane_Unigene_BMK.60969, gi36066432. A *ScChi*VII1 sequence was isolated by homology-based cloning method according to the predicted chitinase gene (XM_002460419.1) from *S. bicolor* genome database.

According to the results of previous researches by Kirubakaran *et al.*^28^, Rahul *et al.*^33^ and Singh *et al.*^17^, along with the information from bioinformatic analysis and their expression profile under the stresses of MeJA, ABA and SA indicated in this study, three out of ten chitinase genes, *ScChi*I1, *ScChi*IV1 and *ScChi*VII1 were chosen for further study. Based on the sequences of the above predicted chitinase genes, the primers used to clone the target genes were designed. Amplification of *ScChi*I1 (gi32815041) was performed with primers *ScChi*I1: FW-ACATACATAGTTGCTTGCYTTGC and RV-CCTTTTGCTTTATTCATTGCTC on first-strand cDNA template of Yacheng05-179 under 4 °C low temperature treatment for 24 h. *ScChi*IV1 (Sugarcane_Unigene_BMK.56580) and *ScChi*VII1 were amplified with primers *ScChi*IV1: FW-GCACCGCAGCAACGAA and RV-CGGAGCCATGCAAGGAG, *ScChi*VII1: FW-AAGATGAAGCGGAAGACG and RV-GCTAAAACAGACCCATTGTG, on first-strand cDNA template of Yacheng05-179 post 48 h *S. scitamineum* inoculation. These PCR products were gel-purified, cloned into the pMD18-T vector (TaKaRa, China) and sequenced (Shenggong, China).

### Sequence analysis of chitinase genes

ORF analysis was performed with the ORF Finder (http://www.ncbi.nlm.nih.gov/gorf/gorf.html). The pI was calculated with the ProtParam tool (http://www.expasy.ch/tools/protparam.html). SignalP 4.0 Server (http://www.cbs.dtu.dk/services/SignalP/), NCBI Conserved Domains (http://www.ncbi.nlm.nih.gov/Structure/cdd/wrpsb.cgi) and SMART (http://smart.embl-heidelberg.de/) programs were employed to scan for the signal peptides and the motifs on the primary structure of the deduced protein sequences. Subcellular location of the putative proteins was predicted with PSORT Prediction (http://psort.hgc.jp/form.html). ClustalW software was used to perform multiple alignment of sugarcane chitinases with other previously published plant chitinases^17^,^18^. Based on this alignment, a phylogenetic tree was constructed according to the neighbor-joining (NJ) method (1,000 bootstrap replicates) using the MEGA 5.05 program.

### Transcript level analysis

Expression patterns of sugarcane chitinase family genes in different tissues and their response to biotic and abiotic stress were analyzed by qRT-PCR, which followed the instructions of the SYBR Green Master (ROX) (Roche, China) on a 7500 real time PCR system (Applied Biosystems, USA). The *GAPDH* (glyceraldehyde-3-phosphate dehydrogenase) gene (Table 2) was used as an internal control. According to sequences of *ScChi*I1* ~ ScChi*VII1, the specific primers (Table 2) were designed using the Beacon Designer 8.12 program. The qRT-PCR reaction system (20 μL) contained 10 μL FastStart Universal SYBR Green PCR Master (ROX), 1.0 μL of first-strand cDNA (10 × diluted) and 0.5 μM of each primer. PCR with distilled water as template was performed as control. The qRT-PCR reaction condition was held at 50 °C for 2 min, 95 °C for 10 min, 40 cycles of 95 °C for 15 s and 60 °C for 1 min. At the end of the PCR reaction, a melting curve was established. Each qRT-PCR was conducted in triplicate. The 2^−ΔΔCt^ method was adopted to analyze the qRT-PCR results^53^. For calculating gene expression level during developmental stages, the tissue exhibiting the lowest expression level was served as control. For the abiotic stress treatments, unstressed sample was used as control. During the biotic stress, gene expression profile was calculated by the expression level of the inoculated sample of *S. scitamineum* minus the level of the mock at each corresponding time point to eliminate any effect of wounding. Data points in qRT-PCR time course were plotted as means ± SE of three replicates.

### The role of three chitinase genes in response to pathogen infection

Subcellular location assay with *Agrobacterium*-mediated transformation was followed from Su *et al.*^22^. ORF fragments of *ScChi*I1, *ScChi*IV1 and *ScChi*VII1 were inserted into the vector of pCAMBIA 2300-GFP and transformed into the competent cells of *A. tumefaciens* strain EHA105, respectively. The subcellular localization of the fusion protein was visualized using a confocal laser scanning microscope Leica TCS SP5 (Germany) equipped with 10 × lense.

As reported, cell death presented at the infected site is the most efficient method to restrict pathogen growth and development^54^. The stimulation of reactive oxygen species (ROS) and defense-related hormones, induction of R gene expression and ion fluxes are the common response of cell death^55^,^56^. For the transient expression of the target gene in *N. benthamiana*, overexpression vectors pCAMBIA 1301-*ScChi*I1, pCAMBIA 1301-*ScChi*IV1 and pCAMBIA 1301-*ScChi*VII1 were constructed to analyze their defense responses. *Agrobacterium* strain EHA105 carrying the recombinant vector was transiently expressed in *N. benthamiana* leaves. Each treatment was carried out in three replicates. DAB (3,3’-diaminobenzidinesolution) was used to stain H_2_O_2_ produced in agroinfiltrated leaves^22^. The leaves were incubated in 1.0 mg/mL DAB-HCl solution in the dark overnight and destained by boiling in 95% ethanol for 5 min. The bronzing color of the leaves for H_2_O_2_ detection was photographed. qRT-PCR analysis of the expression of seven immunity associated marker genes were conducted post 24 infiltration, including the hypersensitive response marker genes *NtHSR201* and *NtHSR203*, the jasmonate associated genes *NtPR-1a/c*, *NtPR2* and *NtPR3*, and the ethylene synthesis depended genes *NtEFE26* and *NtAccdeaminase* (Table 2)^22^. *NtEF1-α* (Table 2) was used to normalize the transcript levels.

## Additional Information

**How to cite this article**: Su, Y. *et al.* Identification, Phylogeny, and Transcript of Chitinase Family Genes in Sugarcane. *Sci. Rep.* **5**, 10708; doi: 10.1038/srep10708 (2015).

## Supplementary Material

Supporting Information

## Figures and Tables

**Table 1:** A list of differentially expressed chitinase genes in Yacheng05-179 and ROC22 after challenged with *Sporisorium scitamineum* for 24 h, 48 h and 120 h, respectively.

**Unigene ID**	Yacheng05-179 log_2_ fold change (T/CK)*			ROC22 log_2_ fold change (T/CK)*			**BLAST annotation**
	**24 hpi**	**48 hpi**	**120 hpi**	**24 hpi**	**48 hpi**	**120 hpi**	
gi32815041	—	—	—	—	—	1.64	Chitinase 1 [*Oryza sativa*]
gi34957207	—	2.38	—	—	−2.14	−1.88	Chitinase 12 [*Oryza sativa*]
Sugarcane_Unigene_BMK.68059	1.49	—	—	—	—	—	Chitinase 12 [*Oryza sativa*]
gi35992663	1.47	—	1.22	—	1.68	4.23	Chitinase 11 [*Oryza sativa*]
Sugarcane_Unigene_BMK.51590	—	—	—	−1.55	—	—	Acidic endochitinase [*Vitis vinifera*]
Sugarcane_Unigene_BMK.60821	1.37	—	—	—	—	2.34	Acidic endochitinase [*Vitis vinifera*]
Sugarcane_Unigene_BMK.56580	1.97	2.10	—	—	—	1.55	Endochitinase [*Zea mays*]
Sugarcane_Unigene_BMK.64954	—	1.70	—	—	—	—	Chitinase 2 [*Tulipa bakeri*]
Sugarcane_Unigene_BMK.48857	1.61	1.68	—	—	—	—	Chitinase 10 [*Oryza sativa*]
gi36021860	1.61	1.68	—	—	—	—	Endochitinase A [*Zea mays*]
Sugarcane_Unigene_BMK.60821	1.37	—	—	—	—	—	Acidic endochitinase [*Vitis vinifera*]
gi35081719	—	—	—	—	1.33	—	Chitinase 6 [*Oryza sativa*]
Sugarcane_Unigene_BMK.49423	—	—	—	—	—	1.64	Chitinase 1 [*Oryza sativa*]
gi36003099	—	—	—	—	—	3.60	Chitinase 1 [*Tulipa bakeri*]
gi35980761	—	—	—	—	—	2.08	Chitinase 2 [*Oryza sativa*]
Sugarcane_Unigene_BMK.60969	—	—	—	—	—	1.87	Chitinase 2 [*Oryza sativa*]
gi36066432	—	—	—	—	—	1.96	Chitinase 8 [*Oryza sativa*]

^*^T indicated the transcriptome of sugarcane challenged with *S. scitamineum*. CK mean the transcriptome of the mock material.

**Table 2:** qRT-PCR primers used in this study.

**Primer**	**Forward primer (5’-3’)**	**Reverse primer (5’-3’)**
*ScChi*I1	TTGCTTTGCTTCCCTCACGA	AGAGGGACTCGGAGATGATGGA
*ScChi*I2	CCTGTTCAACCAGATGCT	AAGGCGGAGTAGGTGTAG
*ScChi*I3	GTCCTCACCAACATCATCA	GCTGTAGCAGTCCAAGTT
*ScChi*II1	TGACCACCAACATCATCAA	ATTCCAAGCATATCGCAGTA
*ScChi*III1	CATCAAGGTCCTGCTCTC	CCGAGGTAGTTGTTCCAG
*ScChi*III2	ATGGCGGCTAATCTCAAG	GATGACGTAGGCGTAGAG
*ScChi*IV1	CGAGACCGGACATTTCTGCTAC	CGAACCCCTGCGACATCAC
*ScChi*V1	GACGAGCAGATGGTGAACT	CCAGATGAAGATGCCGTAGAG
*ScChi*VI1	GTGGTCGCTTTCCTCGTTGC	TGGATGAAGGAGGCGTAGGTGT
*ScChi*VII1	TCAAGAAGAACCAGCCGTCAGC	TGCTGCTTCTGGTCATCCGTTG
*GAPDH*	CACGGCCACTGGAAGCA	TCCTCAGGGTTCCTGATGCC
*NtHSR201*	CAGCAGTCCTTTGGCGTTGTC	GCTCAGTTTAGCCGCAGTTGTG
*NtHSR203*	TGGCTCAACGATTACGCA	GCACGAAACCTGGATGG
*NtPR-1a/c*	AACCTTTGACCTGGGACGAC	GCACATCCAACACGAACCGA
*NtPR2*	TGATGCCCTTTTGGATTCTATG	AGTTCCTGCCCCGCTTT
*NtPR3*	CAGGAGGGTATTGCTTTGTTAGG	CGTGGGAAGATGGCTTGTTGTC
*NtEFE26*	CGGACGCTGGTGGCATAAT	CAACAAGAGCTGGTGCTGGATA
*NtAccdeaminase*	TCTGAGGTTACTGATTTGGATTGG	TGGACATGGTGGATAGTTGCT
*NtEF1-α*	TGCTGCTGTAACAAGATGGATGC	GAGATGGGGACAAAGGGGATT
