# Supplementary material for: Identification, Phylogeny, and Transcript of Chitinase Family Genes in Sugarcane
Source: Sci Rep. 2015 Jun 2;5:10708. doi: 10.1038/srep10708 (PMC4451799; doi:10.1038/srep10708)
Supplement: Supporting Information [file srep10708-s1.doc]

**Identification, phylogeny, and transcript of chitinase family genes in sugarcane**

Yachun Su, Liping Xu*, Shanshan Wang, Zhuqing Wang, Yuting Yang, Yun Chen & Youxiong Que*

Key Laboratory of Sugarcane Biology and Genetic Breeding, Fujian Agriculture and Forestry University, Ministry of Agriculture, Fuzhou 350002, China

*Correspondence and requests for materials should be addressed to L.X. ([xlpmail@126.com](mailto:xlpmail@126.com)) or Y.Q. ([queyouxiong@126.com](mailto:queyouxiong@126.com))

**Supplementary information**

**Table S1** List of the nucleotide and protein sequences of the ten sugarcane chitinase genes

| Gene name | Nucleotide sequences | Protein sequences |
| --- | --- | --- |
| *ScChi*I1 | TGCTTGCTTTGCTTCCCTCACGATGATGAGAGCACTAGCGGTGGTGGCCATGGTGGCCACTGCTGCCTTGCTCGCCGCGTCCACGCGTGCCGAGCAGTGCGGCACGCAGGCCGGCGGCGCGCTGTGCCCCAACTGCCTATGCTGCAGCAAGTTCGGGTGGTGCGGCACCACCTCCGACTACTGCGGCAGCGGGTGCCAGAGCCAGTGCACCGGCAGCTGCGGCAGCACTCCCACTCCCACCCCGAGCTCCGGCGGTGGCAGCGTGGCGTCCATCATCTCCGAGTCCCTCTTCAACCAGATGCTCCTCCACCGCAACGACGCGGCGTGCCCAGCCAAGGGCTTCTACACCTACGCCGCCTTCATCGCAGCGGCCAAGAAGTTCCCGGGCTTCGGCACCACGGGCGGCGCCGACACGCAGAAGCGGGAGCTGGCGGCGTTCCTAGCCCAAACCTCGCACGAGACGACGGGCGGGTGGGCGACGGCGCCCGACGGCGCCTACGCCTGGGGCTACTGCTTTAAGGAGGAGCAGGGCGCCGCGGCGGGGCCGGACTACTGCAAGCCCAGCACGCAGTGGCCGTGCGCCGCAGGGAAGAAGTACTACGGCCGTGGGCCCATCCAGATCTCCTACAACTACAACTACGGCCCCGCGGGGCAGGCCATCGGCGCCGGCATCCTCGCCAACCCGGACCTGGTGGCGTCGGACCCCACAGTGTCATTTGAGACGGCCGTCTGGTTCTGGATGACGCCGCAGTCGCCCAAGCCGTCGTGCCATGCCGTCATGACCGGGCAGTGGACGCCCTCCGGAGCTGACACCGCCGCCGGAAGGCTGCCGGGGTATGGTGTCGTCACTAACATCATTAATGGTGGCCTCGAGTGCGGCCATGGCGCTGATAGCCGCGTTGCCGACCGGATTGGGTTCTACAAGCGCTACTGCGACTTGCTTGGGGTCAGCTACGGCGACAACTTGGACTGCGCCAACCAGAGGCCCTTCAACAGCTAGATCGATGAACAAAAATCAGAGCATGCATGCAGTCTTATGAGTTACATACGTACGTACTATATATGATGAATTACCAACACAATAA | MMRALAVVAMVATAALLAASTRAEQCGTQAGGALCPNCLCCSKFGWCGTTSDYCGSGCQSQCTGSCGSTPTPTPSSGGGSVASIISESLFNQMLLHRNDAACPAKGFYTYAAFIAAAKKFPGFGTTGGADTQKRELAAFLAQTSHETTGGWATAPDGAYAWGYCFKEEQGAAAGPDYCKPSTQWPCAAGKKYYGRGPIQISYNYNYGPAGQAIGAGILANPDLVASDPTVSFETAVWFWMTPQSPKPSCHAVMTGQWTPSGADTAAGRLPGYGVVTNIINGGLECGHGADSRVADRIGFYKRYCDLLGVSYGDNLDCANQRPFNS |
| *ScChi*I2 | GATGCATCCACGCGTTCGGGATCCTAGAGGTAGCTAGCTTCTCGTTGTTGTTTCCCATGCCCATGCCCATGCCCATGGTCATGACGACAAGAGCACTGGCAGTAGTGGCCATGTTGGCCACGGCGTTCGCCGAGCAGTGCGGCTCGCAGGCCGGCGGCGCGCTCTGCCCCAACTGCCTCTGCTGCAGCAAGTGGGGCTGGTGCGGCACCACCGCCGACTACTGTGGCGATGGCTGCCAGAGCCAGTGCAGCGGCAGTTGCGGCGGCACCCCGCCCCCACCCCCTCCCCCTCCCACTCCCACCCCTAGCCCCCCGAGCGGCGGTGGCGTGGCGTCCATCGTCTCCGAGTCCCTGTTCAACCAGATGCTGCTGCATCGGAACGACGCCGCGTGCCCAGCCAACGGCTTCTACACCTACTCCGCCTTCATTGCGGCGGCGGACGCGTTCCCGGGCTTCGGCACCACGGGCGGCGCCGACGTCCAAAAGCGCGAGCTGGCGGCGTTTCTGGCGCAGACGTCGCACGAAACGACGGGCGGGTGGGAGACGGCGCCAGAAGGCCCCTACGCCTGGGGCTTCTGCTTTAAAGAAGAAGTCGGGCGGGTGCAAGGGCCCGACTACTGGCAACCCATAACCCAGTGGGCATGCCCCGTTGGCAAAAAGTCCTTCGGGCGCGGGCCCATCCAGCTTGGCTGGAACTACAACTACGGGCCCGCCGGGGGGGCCATTGGGAAGGACCTTTTAAATAACCCCTGTTTTGTGTTGCCGGGAAGTTAAAAATCGTCTTTTAGAAACGGGCTTTTGGTACTGGATTAACACACGAACCGCTCCTAACCCTTTTTTGTCAAAAGCTAATGAAGGGCTCATGTGAATGCCCATTGCCCTACATTTTAGCCTGTGGGAAGAATGATAGAATATTGCGTTACCATAAAAATTTTAAAAGGGTGAGTTTTGTATGTGCACTCTGTATATTAATGTCTGTATTATTGTGTTGAGTTATTGCTTAACT | MPMPMPMVMTTRALAVVAMLATAFAEQCGSQAGGALCPNCLCCSKWGWCGTTADYCGDGCQSQCSGSCGGTPPPPPPPPTPTPSPPSGGGVASIVSESLFNQMLLHRNDAACPANGFYTYSAFIAAADAFPGFGTTGGADVQKRELAAFLAQTSHETTGGWETAPEGPYAWGFCFKEEVGRVQGPDYWQPITQWACPVGKKSFGRGPIQLGWNYNYGPAGGAIGKDLLNNPCFVLPGS |
| *ScChi*I3 | CCCAATGTCTTCCTTAGATGGGGAAGCAGCAGCGAGCACGGGTTTCTGTCGCGAGCGCGTGCACAGAAAACTTGCAGAAATGGTGATTCAAGGGCTTGGACCGGGACCCTTAGGAATGAGGGGTGGGGGATTTAAGGGTCCTCTCAAATTTAAGGGCTCTAGTAGGGTCCTATTAGAATGCAGTTTTTACCTCTCAGCCTAAAAAATAGGATGTGAGAGCTCAAATGGGGTTTTGATGAATTTACTTTAAGAATATAAAAAACGGAGTCGATCAATAACCACTTAATTATTATTTTATTTCATAAACTAATTCGACAACATTTTGCATTCTTTTTCACACGCTCGATCTGACTCGATCATTACTGGTGTACTTAGTCTGAGCTCTGCCTTGTAGTCGTAGCAGCTCTCTTCTCGTGCCCATTACTTAGTTTACGCCTCAGCATGATATGAGGTAGCAGTAGTTTCAGCTAAGATTGTGTTTCCGAAGGGCCTCTGGCTGTAGCAGTCCAAGTTGTCGCCGTAGCTGACACCAAGCATGTCGCAGTAGCGTTTGTAGAAGCCGATCCGGCTGGCAACACGGTCGTCGGCGCCGCGGCCGCACTCGAGGCCGCCGTTGATGATGTTGGTGAGGACGCCATATCCCGGCAGCCTCCCCGCGGCCACATCGGCGGGCGAGGGCGTCCACTGGCCCGTGATGGCGTCGTGGCAGGACGGCTTGGGCGGCTGCGGCGTCATCCAGAACCATAGCGCCGTCTCGAAGGCGATCTTGACGTCGCCGGCAACCAGACCAGGGTTGTTGAGAAGGTCCTCGCCGATGGCCTCCCCGGCGGGCCCGTAGTTGTAGTTCCAGGACAGCTGGATGGGCCCGCGGCCGTAGTACTTCTCGCCAGCGGCGCATGGCCACTGGGTGCTGGGTTGGCAGTAGTCCGGCCCCTGCACGCCGCCGACCTCCTCCTTGAAGCAGTAGCCCCAGGCGTAGGGGCCGTCTGGCGCCGTCTCCCACCCGCCCGTCGTTTCGTGCGACGTCTGCGCCAGGAACGCCGCCAGCTCGCGCTTTTGGACGTCGGCGCCGCCCGTGGTGCCGAAGCCCGGGAACGCGTCCGCCGCCGCAATGAAGGCGGAGTAGGTGTAGAAGCCGTTGGCTGGGCACGCGGCGTCGTTCCGATGCAGCAGCATCTGGTTGAACAGGGACTCGGAGATGATGGACGCCACGCCACCGCCGCTCGGGGGGCTAGGGGTGGGAGTGGGAGGGGGAGGGGGAGGGGGAGCAGGAGCATCCACGAGAGGAGAGCGAAGGAGATTCCCATTCGCCCCCATCCGTCTCTCATCTCACCGGAAGCGAAGCCGGAGGAGGCGGAGGAGGGAGAGAGGAGATCGCGAGCAGCCGGAGCCGGAGGCCGAGAAGATGAAGACGTTCGACCCGTGGCCGGTCTTCTTCCGCCGGGAGTGGAGGCGCAACTGGCCCTTCCTCACGGGGTTCGCCATCACCGGATTCCTCATCACCAAGATGACGGCCAACTTCACCGAGGAGGACCTCAAGAACTCCAAGTTCGTCCAGGAGCACAAGAGGCACTGACCGACCAGGCGGAGTCGTCCGAATTGCCTGTGTGGACAGCCA | MGANGNLLRSPLVDAPAPPPPPPPTPTPSPPSGGGVASIISESLFNQMLLHRNDAACPANGFYTYSAFIAAADAFPGFGTTGGADVQKRELAAFLAQTSHETTGGWETAPDGPYAWGYCFKEEVGGVQGPDYCQPSTQWPCAAGEKYYGRGPIQLSWNYNYGPAGEAIGEDLLNNPGLVAGDVKIAFETALWFWMTPQPPKPSCHDAITGQWTPSPADVAAGRLPGYGVLTNIINGGLECGRGADDRVASRIGFYKRYCDMLGVSYGDNLDCYSQRPFGNTILAETTATSYHAEA |
| *ScChi*II1 | AAGTAAGCCAGCCATGGCGAGGGCTTTGTCGACGGTGCTGGTCCTGGCAGGCGCCGCGCTGCTCGCCGCAGCCGGTAGCGGCGCCAGCGCGCAGCAGGGCGTGTGGAGCATCATCACGCGGCCCATGTTCCAGAGCATGCTGAGCCACCGCGGCGACAGCGGCTGCCAGGGCGCCTTCTACACCTACGACGCGTTCATCGAGGCCGCCAGCAAGTTCCCCGGCTTCGGCACCACCGGCGACGACCAGACGCGCAGGCGGGAGCTCGCCGCCTTCTTCGGCCAGACGTCCCACGAAACCACCGGTGGATGGGCAACTGCTCCGGGTGGACAGTTTGCCTGGGGATATTGCCGGGTGAAGGAACAGAACCCGACTGACCCACCCTACTATGGACGAGGACCCATACAGCTAACTCATGAGTACAACTACAGGCAAGCTGGGCAAGCGCTGAAGCTGAACCTGGTGGGCAACCCGGACCTGGTGTCGAGCGACCCCGTGGTGGCTTTCAAGACGGCCATCTGGTTCTGGATGACGCCGCAGTCGCCGAAGCCGTCGTGCCACGCCGTCATGACCGGCGGNTGGACGCCCTTCGCCGNCGACCGCGCCGNGGGGAGGCTCCCCGGGTATGGCGTGACCACCAACATCATCAACCGCGGGGCTGGAGTGCGGGGAAGGCCAGTCCAACGACGGCGCCAAGGGACCGGGTCGGCTACTACCAGAAGTACTGCGATATGCTTGGAATGGGGTACGGGGACAAACTGTCCTGCAAGGACCAGAAGCCTTACGGAGGGTGGCAACAATGGAACAAAAAATGCTGTAATTAAATTTTGTTGCTGGCAAACTCTACAGTGGTTGGCTTTTTTGCTTTAACTAGTGGAAAGAATAAATTAGCTTGCAAAAAGGGGGAACCTAAGTTTGGCCCGGGGTTACTTTTAATGAAAATATTTTTCAGAAGGAATACGTTACCATCAAAAAAAAAAAAAAAGGGGGGCGGTTCTTAAGGATCAGATTTAAAATACGGGATGTCGA | MARALSTVLVLAGAALLAAAGSGASAQQGVWSIITRPMFQSMLSHRGDSGCQGAFYTYDAFIEAASKFPGFGTTGDDQTRRRELAAFFGQTSHETTGGWATAPGGQFAWGYCRVKEQNPTDPPYYGRGPIQLTHEYNYRQAGQALKLNLVGNPDLVSSDPVVAFKTAIWFWMTPQSPKPSCHAVMTGGWTPFADRAGRLPGYGVTTNIINRGAGVRGRPVQRRRQGTGSATTRSTAICLEWGTGTNCPARTRSLTEGGNNGTKNAVIKFCCWQTLQWLAFLL |
| *ScChi*III1 | CAGAACTGCGCCGCTGCTAGTTGCCTACCACGGATTCATCATCAGCTATATAAAGACCAGGGATCGACCTTCAAGCACAATTCATACTCACTAGTCCATACCAAGAACTCGAGAAACAAGATGGCTAGAAGCAACATCCCGTCTCTGCCAATGCTGTTGGCCACGGCGTTTTCATTGGCCGGGGTAGCCGCCGCCGGAGCGCGCGCCGGCGGCATCGCCATCTACTGGGGCCAGAACGGCAACGAGGGGACGCTGGCGGAGACCTGCGCCACGGGCAACTACAAGTTCGTCAACGTGGCGTTCCTCCCGACGTTCGGCAAGGGCCAGACGCCGGTGCTGAACCTGGCGGGCCACTGCGACCCGGCGAGCAACGGGTGCACGGGCGTGGGCGCGGACATCAAGGCGTGCCAGCGCAGGGGCATCAAGGTCCTGCTCTCCATCGGCGGGGGCGTCGGCAGCTACGGGCTCTCGTCCCCGGACGACGCGAGGAGCGTCGCGGCGTACCTCTGGAACAACTACCTCGGCGGCAGGTCGTCCAGCACCAGGCCCCTCGGCGACGCCGTCCTCGACGGCATCGACTTCGACATCGAGAGCGGCGGGAGCTTGTACTGGGACGATCTGGCGAAAGCCCTCAAGTCCTACTCCCGGCGCCGCGGGCGGAAGCCGGTGTACCTGTCGGCGGCGCCGCAGTGCCCGTTCCCGGACGCGTCGCTGGGCACGGCGCTCGGCACGGGGCTGTTCGACTACGTGTGGGTGCAGTTCTACAACAACCCGCCGTGCCAGTACAGCGCGAGCGCCGGCGTGGGCAGCCTGGCGAGCGCGTGGGCGCAGTGGACGTCCATCAGGGCCGGGCGGGTGTTCCTCGGCCTCCCGGCCGCGCCACAGGCCGCAGGCAGCGGGTTCGTGCCGGCGACCGACCTCGTGTCGCAGGTGCTGCCGGTGGTGAAGAACTCCACCAAGTACGGGGGCATCATGCTCTGGTCCAGGTACTACGACGGGCTCACGGGGTACAGCGACGCGGTCAAGTCCCAAGTGTGAGCTAGGCAGGCCCTCGTGTCATGTCGACCTGGTCTGGGATGCACATGCGTCCATGTGCCTATAGTATGTGCGTGCGGGGCGGTGTACTGACGTTGCTTAAAGAAGCGACGGGAAAAAAAGAGCAAGTCGCAGATAGTGCTTCTTACGTACTTGTTAAACCATTTTTCACTTACTCTTTCGGGTGAACACCCATTTGATGTTTCAAGCAAAATAAAAAAAAATAGAGTATATTCAGAGAA | MARSNIPSLPMLLATAFSLAGVAAAGARAGGIAIYWGQNGNEGTLAETCATGNYKFVNVAFLPTFGKGQTPVLNLAGHCDPASNGCTGVGADIKACQRRGIKVLLSIGGGVGSYGLSSPDDARSVAAYLWNNYLGGRSSSTRPLGDAVLDGIDFDIESGGSLYWDDLAKALKSYSRRRGRKPVYLSAAPQCPFPDASLGTALGTGLFDYVWVQFYNNPPCQYSASAGVGSLASAWAQWTSIRAGRVFLGLPAAPQAAGSGFVPATDLVSQVLPVVKNSTKYGGIMLWSRYYDGLTGYSDAVKSQV |
| *ScChi*III2 | CGGAGCTGAAGCATGCCACACATTTGAGCCAAAAAGTTATGGCGACTGCTTCTCCCAACTATGGCGAGCCTTACTTTGATAATGAGTCAAACGCCAGTCACAACACCTGTGGCGAGCCTAACATTAGTCGTGGCGAGTTGTGACGGGCAACCAAGCATTGGTTAAGCATTGGTGCGCGTTGTTCTCGTCAAGGCCCTACGACCACGAGAGAATAGCTGACATGCGTTCAATGCTGCCAGAAATGCATGTGTTGTGGTCATTTTGTACTTTTGACCGGAGGGTATTGCAAGTCAAGTTTTATTCATTGGGTATATATAACACAAACTTATCGTAGAATTGTGTGGTGTCGTGTTCGTCCGTGCATATATTAGCTACTCGCTACTGTAGTGTGTGTAGCAGATGCATCTTTGTCCCCTGATGATCCGCTCTTATTGTATCTAGTCCGATAACTTGACGAATGAATAGCCACTGTATCTGTATGGCTACCTCTGCTGTAGCCTGAAGCTTCGTATGCTCAGACGCTGCCCTTCACCTGGGCGCTGTAGTTGTTCTGCACGTCGTAGAAGCGGTTCCACAGCATGATGCCGCCGTACTTGCCAACGCCCTGGATGGCGGGGATCACCGTGCCCGTCAGCGTGCCGGGATCGATGTACCCGCTGCCGGCGGCCTGGGTCGACGCCGGCACGCCGAGGTAGAAGCTCCCCGCCGTGACGCTGCTGGTCCACGTGCTCCAGGCGTTCACCAGGTTGGTGTCGTCCCCGTTGGCGTACGCGCACCCGGGGTTGTTGTAGAACTGGATCCACACGTTGTCGAACTGCCCCGTCTGCAGCGCGGGCCCCAGCGACGCGTCCGGGTAGGGGCACTGCGGCGCCGCCGACAGCAGCACGCTCCCCTTGCCCTTGAGCGCGTTCGCCAGGTCGTCGTAGTGCGCCGACTGCCCGTTCTCGATGTCGAAGTCGACGCCGTCGAGCACGGCGGCGCCGAGTGGGCGGGTCGAGCCGTCGCTGCCGCCCAGGTAGCTGTTCCACAGGTAGTCGGCCACGCTGTTGGCGTCGTCGGTGGAGGAGAGGCCGTAGCTGCCGCTGCCGCCGCCGATGGAGAGGAGCACCTTGATGCCCTGCGACTGGCAGGTCTGGATGTCGCTGCTCAGGCCGGTGCAGCCGCCGGAGCCCGGGTCACAGTGCCCGGCGAGGTTCAGGACCGGGGTCTGTCCGTTGCCGAAGGTTGTGAGGAACGCGAGGATGACGTAGGCGTAGAGCCCGGAGTTGCAGGTGTCCGCCAGGGTGCCTTCGTTGCCGTTCTGGCCCCAGTACACCGCGATATTGCCAGCACTGGTGCTGCCAACCATGGCAGCAACGAGGAGAAGGGCCAGAACAGGAGGCCACTTGAGATTAGCCGCCATGTTGCTTACTGGTCTTGATGATGAGACGAGGAAGCTGCTTCAGTTGCTGGTTCAGAGGACCAGCTACTTGCTTTTTGGCTTGGTGCCTTGGTTGGTGG | MAANLKWPPVLALLLVAAMVGSTSAGNIAVYWGQNGNEGTLADTCNSGLYAYVILAFLTTFGNGQTPVLNLAGHCDPGSGGCTGLSSDIQTCQSQGIKVLLSIGGGSGSYGLSSTDDANSVADYLWNSYLGGSDGSTRPLGAAVLDGVDFDIENGQSAHYDDLANALKGKGSVLLSAAPQCPYPDASLGPALQTGQFDNVWIQFYNNPGCAYANGDDTNLVNAWSTWTSSVTAGSFYLGVPASTQAAGSGYIDPGTLTGTVIPAIQGVGKYGGIMLWNRFYDVQNNYSAQVKGSV |
| *ScChi*IV1 | GCACCGCAGCAACGAACCTAGCTCAGTCAGAAGCTGAAGTACTGATCCAGTATGGCAAACGCGCCGAGGATCTTGGCGGTCCTGGCTCTCGGGCTAGCGCTCCTCTGCGCTGCCGGCCCGGCCGCCGCGCAGAACTGCGGCTGCCAGCCAAACTACTGCTGCAGCAAGTTCGGATACTGCGGCACGACCGACGACTACTGTGGCGACGGGTGCCAGTCGGGCCCGTGCCGCTCGGGCGGCGGTGCCAGCAGTGGTGGTGGGAACGTGGCTAGCGTTGTCACCGACGCATTCTTCAACGGCATCAAGAACCAGGCCGGGAACGGGTGCGAGGGCAAGAACTTCTACACCCGGAGTGCGTTCCTGAGCGCCGCCGACTCGTACAAAGGCTTCGGTGGCGTGTCGGTGGAGGGCAAGCGCGAGATCGCCGCCTTCTTCGCGCACGTCACGCACGAGACCGGACATTTCTGCTACATCAGCGAGATCAACAAGAACAACGCCTACTGCGACGCGAGCAACAGGCAGTGGCCGTGCGCCGCGGGACAGAAGTACTACGGGCGCGGCCCGCTGCAGATCTCGTGGAACTACAACTACGGGCCTGCCGGGAGGGACATCGGCTTCAACGGGCTCGGGGACCCCAACAGGGTGGCGCAGGACGCCGTGATCGCGTTCAAGACGGCGCTCTGGTTCTGGATGAACAACGTGCACCGGGTGATGTCGCAGGGGTTCGGAGCCACCATCAAGGCTATCAACGGAGCCCTCGAGTGCAACGGAAACAACCCCGCCCAGATGAACGCGCGGGTGGGCTACTACAAGCAGTACTGTCAGCAGCTCGGCGTCGACCCGGGGCCCAACCTCACTTGCTAGGCGACTGGCGTCGGCGAGTCCTTGGCTCCTTGCATGGCTCCG | MANAPRILAVLALGLALLCAAGPAAAQNCGCQPNYCCSKFGYCGTTDDYCGDGCQSGPCRSGGGASSGGGNVASVVTDAFFNGIKNQAGNGCEGKNFYTRSAFLSAADSYKGFGGVSVEGKREIAAFFAHVTHETGHFCYISEINKNNAYCDASNRQWPCAAGQKYYGRGPLQISWNYNYGPAGRDIGFNGLGDPNRVAQDAVIAFKTALWFWMNNVHRVMSQGFGATIKAINGALECNGNNPAQMNARVGYYKQYCQQLGVDPGPNLTC |
| *ScChi*V1 | TCATCAGTAGACGACACAAACACAAAAACTTGTAGTAGAATGGAAGTAGAATTTCAAGAGCACATGCATTATCTTAACAAAGGGTCACAGCGACATAGCAACTGACAATTTGAGCAAGATTATTTTATTCTCTTGCAACAACAATTTATTCCATATTATTACCGTAATAAAAGGGTAAACAGTTGAAGCAAGGTCCAGTCCAACTCTACTGGCTGGTGGCGTTGGCCAACAGTGCCTGTGCTTGTGTTTCGTACTTGAACCCTTGGCTCTTGGAGTAATCCGCAGCCCAGATGAAGATGCCGTAGAGCTTACCCTGAGACTGCAGGGTCTGGCAGGCGCTGAGCGCCGTATCAACCGGCACCGAGGTCGTCGTCGGCGCCGTGGTGAAGCTGGCCAGGATGTTGCCGCCGGGGTAGTTCACCATCTGCTCGTCGAAGTAGCTCACGTACTGGTCCGCGGTTGTGCTCGCGTCGTACGCGTAGAACTGGAAGTTGATGTAGTCTATCACGCTAATCCCATAGCTCGACCACAGCGCCTGGTAATGGCTCTGCACGTCGGCGTCGGCGAACGGCGCGATGGACGCGAACTTGATCACCCCGTTGCTCTTGAGCGTCGTCACCAGGCGGCCGATGCACTCGGCGAAGGTGGCCGGGTCCGCCTGGAACTGCTCGTAGTCGATGTCGATGCCGTCCAGACCGTACTGCTGGACGATGCTGGTCAGGGAGGACACGGCGTTGTCGACCCACGAGTCGACGGAGGTGACGTTGAAGAACACCGGGCTGTTGTTCACGGTGGCGCCGCCGAGGCTGACGGCCACCCGCACGTTCGGGTTGCTCTGCTTGATGGCGGCCACCGCGGAGGGCGTCAGCACCGAGTCCTGCCAGAAGATGCCGAACTGCCCGTTGGTCGGAGACGGAGGATCGGTTGTGGTGGTGTAGTCGATGACGAAGGAGAGGATGAAGTCCAACTGGACGTCGGGGTTGATCGGCACGTCGCTGAACTGGACACCGTTGAAGATGGCGCCGATGTAGTCGCGGAAGAGGTTGGAGTTTGCTGCGCTGGTCGTTGGGGCATGGAGGAGGGCGATGACAAGGGCAGCAGCTGGGAGAAGAACGGCTGCAATCAGCTTTGAGGAGCCCATTGCTGGTAGTTTTTCTTCTTGTGGACAACCGGTTTGCACTGCT | MGSSKLIAAVLLPAAALVIALLHAPTTSAANSNLFRDYIGAIFNGVQFSDVPINPDVQLDFILSFVIDYTTTTDPPSPTNGQFGIFWQDSVLTPSAVAAIKQSNPNVRVAVSLGGATVNNSPVFFNVTSVDSWVDNAVSSLTSIVQQYGLDGIDIDYEQFQADPATFAECIGRLVTTLKSNGVIKFASIAPFADADVQSHYQALWSSYGISVIDYINFQFYAYDASTTADQYVSYFDEQMVNYPGGNILASFTTAPTTTSVPVDTALSACQTLQSQGKLYGIFIWAADYSKSQGFKYETQAQALLANATSQ |
| *ScChi*VI1 | ATATGCAAAATTCAGAATTATTTGAGAGCAGCAGGTCGTGCCTCTGTGTCATCCGTGGAGCTGAATACCTCCAAATATCCTGCAGGCATCCTGAACGCCGAACTTCTTGCATCTCCCAAGGGCGCAGTAGCAGGTTCGACGATGAACGTGCCGCCGTCCATGAGACGACGACTCCCTTCTCTCTTCGTTGATTGCCGCGCATGGAGGACTAATTTGCATAATTAGCAGTATACAGACGGTTGAGGAGGCAAAGCGTGTAACTCACAGGTGATGTCGTGATTGTCGTCTACTGTTCTTTATTCATATCTGTTTGCATGTATAGGTCAGAACAAGAGAACGTAGGGACACACGTACTACGTTCATCCAAACGTAATATACTTTAATCAACTCTGATAGAAACAGCTTTAAGAACCCCGCGTTAATCAAGTAGTGGCTACCACTAGGCACTAGTACGGCTGCTGGTGTGCGCAGTCGAGGTTGGGGCCGGCGTCGACGTTGAAGATCTGGCAGTACCTCTGGTAGAACCCAATCCTGTTGTTGACCCGGGCATCGTCGGTGCGATTGCACTCGAGCCCGCCGTTGACGATGTTGGTGACGAGCCCGAAGCCGGCCGTCCGGTTGGCCGCGGCGTCGGCGGCCGTCGGGCGGTACTCGCCGACCATGACCTCGTGGCACGACGGCTTGGGCCGGCGCGGCGTCATCCAGAACCACAGCGCCGTCCGGAACGCGGTCTCGGAGCAGTTGGCCACCACCTCCGGGTTGCGCAGGCCGTCGAAGCCCAGCGCCTGCCCCGCCGGCCCGTAGTTGAAGTTCCAGGACAGCTGGATGGGGCCCCGCCCGTGGTAGGACTTGCCCGGGGAGCACGGCCACCGCCTGTCCGTCGCGTCGCAGTAGTTGCTCGCCGGGCTGATCTCCTCCTTGTAGCACAGGCCCCACGCGTACTGGCCGTCCGGCGCCGTCGCCCAACCGCCTGTGGTCTCGTGAGAGATTTGCGCGAAGAAGGCCGCGACCTCGCGCTTGCGGGTGCTGAGGTCGCCCGTGGCGGCGAACTTGGGGAACGTCCTGGCGGCCTGGATGAAGGAGGCGTAGGTGTAGAAGCCCTTGGCGGGGCAGGCGGCGTCGTCCTTGTGCAGGAACAGGGAGTTGTACAGCTGCTCGCTGACGAGGGCCGCCACAGGGGCAGGGGCAGGGCTCCAGTGCCCAGGGCCGTACCTCGCCTCCGCTACGCGGATGCCGACGCTGCCGGAAGCAACGAGGAAAGCGACCACGGCGATCCACGAGGCGCCGTGCACGGCGCAAGAATACGCCATTGTCGTCGACGATGATTGCGATCAAGCTGCGAGTGCCACGAGTGCTCTAGCGCTAGTGCGTGCGTGGGGTTTGTATTGGATTACGGAATGAGTGCTGGAGCGGCCCTCGTCAGCGA | MAYSCAVHGASWIAVVAFLVASGSVGIRVAEARYGPGHWSPAPAPVAALVSEQLYNSLFLHKDDAACPAKGFYTYASFIQAARTFPKFAATGDLSTRKREVAAFFAQISHETTGGWATAPDGQYAWGLCYKEEISPASNYCDATDRRWPCSPGKSYHGRGPIQLSWNFNYGPAGQALGFDGLRNPEVVANCSETAFRTALWFWMTPRRPKPSCHEVMVGEYRPTAADAAANRTAGFGLVTNIVNGGLECNRTDDARVNNRIGFYQRYCQIFNVDAGPNLDCAHQQPY |
| *ScChi*VII1 | AAGATGAAGCGGAAGACGCGGAACAAGATCATCGTGTGGACGCTGGCCCTGGCCGCGGCGGCGATTCTGGTGGGCGGCACGATCGCGCTGGTGCTCACGGCGGGGACGTGGAAGGCCCAGATAAAGAAGTCGCAGGAGAAGATCTGCAACAAGGGGTGGGAGTGCTCCGGGAGCAAGTACTGCTGCAACGACACCATCACCGACTTCTTCAAGGTGTACCAGTTCGAGAACCTCTTCGCCAAGCGCAACACCCCCGTCGCGCACGCCGTCGGGTTCTGGGATTACCGGGCCTTTATCACCGCCGCGGCGCTCTTCGAGCCCCAGGGGTTCTGCACCACCGGCGGCAAGCAGATGCAGATGATGGAGCTCTGCGCGTTTCTCGGGCACGTCGGCGCTAAGACTTCATGTGGGTACGGCGTGGCCACCGGCGGGCCGACGGCGTGGGGGCTATGCTACAACCACGAGATGAGCCCCGACCAGACCTACTGTGACAAGACCTACACCCAGTGGCCCTGCGTTGAGGGTGCCGAGTACTACGGCCGCGGCGCGATACCTGTCTACTGGAACTATAACTATGGCGCCGCAGGCGACGGGATCAAGGTGGACCTGCTCCACCACCCTGAGTACCTCGAGCAGAACGCGACGCTGGCATTCATGGCGGCAATGTGGCGGTGGATGACGCCGATCAAGAAGAACCAGCCGTCAGCGCACGAGGCGTTTGTGGGCACCTGGAAGCCCACCAAGAACGATACGCTAAGCAAACGCCTGCCTGGGTTCGGTGCCACCATGAACATACTCTATGGCGAATCGATCTGTGGCAAGGGATTCATCGATGCCATGAACACTATAATCTCTCACTACCAGTATTACCTTGACCTCATGGGTGTTGGCCGTGAGCACTCTGGCGACAACCGTGATTGTGCTGAGCAGCTCCCGTTCAACCCCTCAAGCCCAACGGATGACCAGAAGCAGCAGCAATCAGGAAGCTAAAACAGACCCATTGTG | MKRKTRNKIIVWTLALAAAAILVGGTIALVLTAGTWKAQIKKSQEKICNKGWECSGSKYCCNDTITDFFKVYQFENLFAKRNTPVAHAVGFWDYRAFITAAALFEPQGFCTTGGKQMQMMELCAFLGHVGAKTSCGYGVATGGPTAWGLCYNHEMSPDQTYCDKTYTQWPCVEGAEYYGRGAIPVYWNYNYGAAGDGIKVDLLHHPEYLEQNATLAFMAAMWRWMTPIKKNQPSAHEAFVGTWKPTKNDTLSKRLPGFGATMNILYGESICGKGFIDAMNTIISHYQYYLDLMGVGREHSGDNRDC |
